# P-366. Investigation of a Bacteremia Outbreak in Dialysis Patients Linked to Closed System Needleless Valve Connectors

**DOI:** 10.1093/ofid/ofae631.567

**Published:** 2025-01-29

**Authors:** Bruna De Souza, Laryssa Aryadne Oliveira, Cinthia Carolini De Jesus, Hoberdan Pereira, Bráulio R G M Couto

**Affiliations:** Hospital Nossa Senhora das Graças, Sete Lagoas, Minas Gerais, Brazil; Hospital Nossa Senhora das Graças, Sete Lagoas, Minas Gerais, Brazil; Hospital Nossa Senhora das Graças, Sete Lagoas, Minas Gerais, Brazil; Hospital Municipal Odilon Behrens, Belo Horizonte, Minas Gerais, Brazil; AMECI – Associação Mineira de Epidemiologia e Controle de Infecções, Belo Horizonte, Minas Gerais, Brazil

## Abstract

**Background:**

Closed system needleless connectors are commonly used in central venous access for long-term intravenous treatments like chemotherapy or dialysis. This paper details an investigation into a sudden rise in bacteremia among dialysis patients.

**Figure 1:**
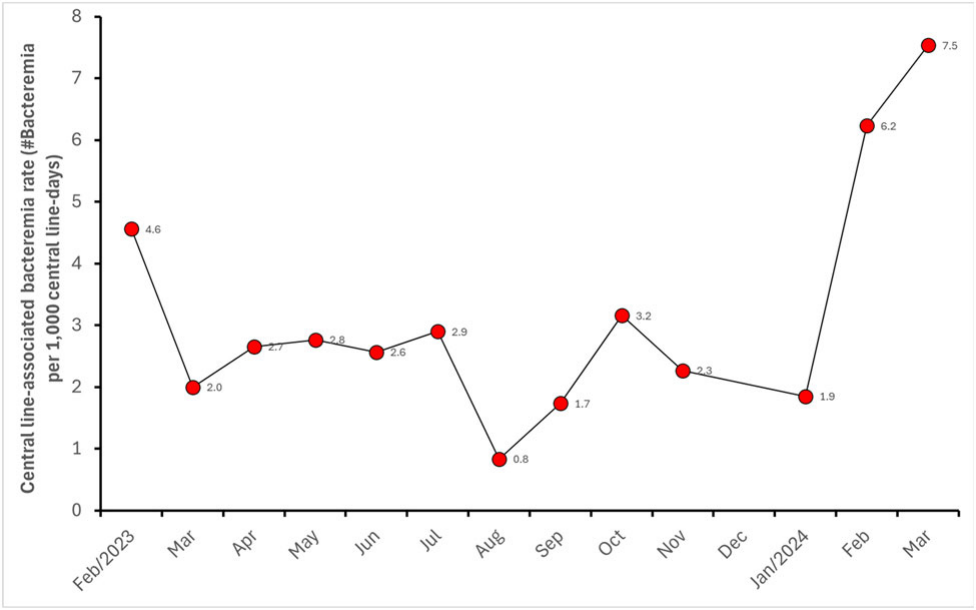
Monthly Rate of Central Line-Associated Bacteremia (#Bacteremia per 1,000 Central Line-Days), February 2023 to March 2024.

Monthly Rate of Central Line-Associated Bacteremia (#Bacteremia per 1,000 Central Line-Days), February 2023 to March 2024.

**Methods:**

Continuous surveillance of hospital-acquired infections (HAIs) in dialysis patients, as prescribed by Brazil's ANVISA. Monthly data analysis revealed an increase in the rate of central line-associated bacteremia in February 2024, which worsened in March (Fig. 1). To define the outbreak, we compared the incidence of bacteremia from the previous period (Feb/2023 to Jan/2024) to the current period (Feb-March/2024). Investigation of the outbreak commenced in February itself.

**Figure 2:**
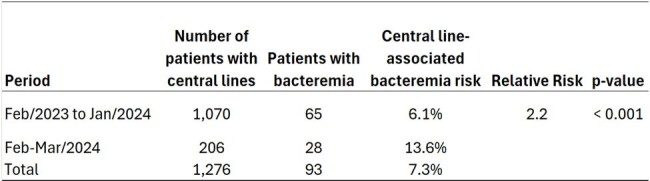
Comparative Analysis of Bacteremia Incidence: Previous Period (February 2023 to January 2024) vs. Current Period (February to March 2024).

**Results:**

The outbreak was clearly defined, with the risk of bacteremia significantly doubling from February to March 2024 (Fig. 2), primarily caused by Staphylococcus species. After interviewing dialysis service professionals about changes in practices, we discovered that a new brand of closed-system valve connectors, purchased from China, began to be used on January 30, 2024. Tests and field evaluations revealed an accumulation of blood in the catheter hub, which could indeed have compromised the integrity of the accesses and facilitated bacterial growth (Fig. 3). The Infection Control and Hospital Epidemiology team suggested discontinuing the use of the device to assess whether there would be a decrease in the number of bacteremia cases. However, due to the high number of devices in stock, it continued to be used throughout the month of March, resulting in a further increase in infection cases. Due to the impact caused by the increase in bacteremia cases, the team decided to suspend the use of all valved devices after the first weeks of April. Consequently, after April 10th, the device was definitively discontinued in the department. The discontinuation of the device resulted in a decrease in the number of infections in April. By April 25th, the total number of bacteremia was 8 cases, of which 6 were reported between April 1st and 10th.

**Figure 3:**
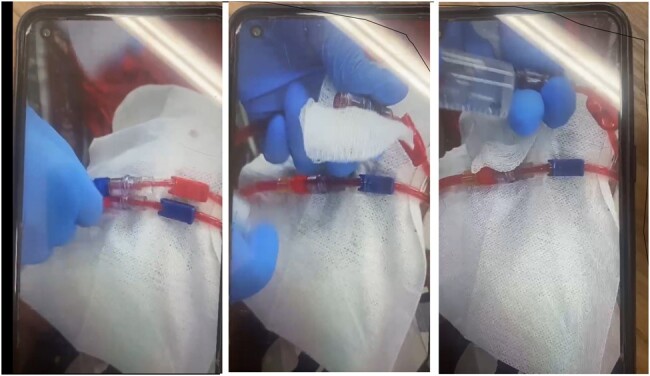
Tests and Field Evaluations Demonstrated Blood Accumulation in the Catheter Hub Using the New Brand of Closed-System Valved Connectors.

**Conclusion:**

It was possible to link the use of the Chinese-made closed-system valved connector to an increase in the number of bacteremia cases, and its discontinuation resulted in a significant reduction in HAIs.

**Disclosures:**

**All Authors**: No reported disclosures

